# Integrating coalescent species delimitation with analysis of host specificity reveals extensive cryptic diversity despite minimal mitochondrial divergence in the malaria parasite genus *Leucocytozoon*

**DOI:** 10.1186/s12862-018-1242-x

**Published:** 2018-08-30

**Authors:** Spencer C. Galen, Renato Nunes, Paul R. Sweet, Susan L. Perkins

**Affiliations:** 1 Sackler Institute for Comparative Genomics, American Museum of Natural History, Central Park West at 79th Street, New York, NY 10024 USA; 2 Richard Gilder Graduate School, American Museum of Natural History, Central Park West at 79th Street, New York, NY 10024 USA; 3 Department of Ornithology, American Museum of Natural History, Central Park West at 79th Street, New York, NY 10024 USA

**Keywords:** Avian malaria, BPP, Cryptic species, GMYC, Haemosporida, UniFrac

## Abstract

**Background:**

Coalescent methods that use multi-locus sequence data are powerful tools for identifying putatively reproductively isolated lineages, though this approach has rarely been used for the study of microbial groups that are likely to harbor many unrecognized species. Among microbial symbionts, integrating genetic species delimitation methods with trait data that could indicate reproductive isolation, such as host specificity data, has rarely been used despite its potential to inform species limits. Here we test the ability of an integrative approach combining genetic and host specificity data to delimit species within the avian malaria parasite genus *Leucocytozoon* in central Alaska.

**Results:**

We sequenced seven nuclear loci for 69 *Leucocytozoon* samples and used multiple species delimitation methods (GMYC and BPP models), tested for differences in host infection patterns among putative species based on 406 individual infections, and characterized parasite morphology. We found that cryptic morphology has masked a highly diverse *Leucocytozoon* assemblage, with most species delimitation methods recovering support for at least 21 separate species that occur sympatrically and have divergent host infection patterns. Reproductive isolation among putative species appears to have evolved despite low mtDNA divergence, and in one instance two *Leucocytozoon cytb* haplotypes that differed by a single base pair (~ 0.2% divergence) were supported as separate species. However, there was no consistent association between mtDNA divergence and species limits. Among *cytb* haplotypes that differed by one to three base pairs we observed idiosyncratic patterns of nuclear and ecological divergence, with *cytb* haplotype pairs found to be either conspecific, reproductively isolated with no divergence in host specificity, or reproductively isolated with divergent patterns of host specialization.

**Conclusion:**

Integrating multi-locus genetic species delimitation methods and non-traditional ecological data types such as host specificity provide a novel view of the diversity of avian malaria parasites that has been missed previously using morphology and mtDNA barcodes. Species delimitation methods show that *Leucocytozoon* is highly species-rich in Alaska, and the genus is likely to harbor extraordinary species-level diversity worldwide. Integrating genetic and ecological data will be an important approach for understanding the diversity and evolutionary history of microbial symbionts moving forward.

**Electronic supplementary material:**

The online version of this article (10.1186/s12862-018-1242-x) contains supplementary material, which is available to authorized users.

## Background

The recent rise in methods to discover and delimit species using multi-locus sequence data under the multi-species coalescent model [1] has given promise to the goal of identifying and characterizing each leaf on the tree of life. Coalescent species delimitation methods have been successfully applied across a broad array of taxa, particularly vertebrates [2–5] and insects [6, 7], though much less often to groups such as microbial symbionts that may be highly diverse yet understudied [8, 9]. Species delimitation has particular relevance for microbial parasites, as infectious diseases often emerge from pathogenic groups for which species limits or evolutionary diversity is poorly known [10–12].

Species delimitation of microorganisms is challenged by a preponderance of cryptic diversity, as microbes typically have limited or highly labile morphological variation that is difficult to characterize using microscopic tools [13–16]. Ecological traits, however, may inform species limits when morphology is not available or reliable [17, 18]. In parasitic groups, for instance, statistical estimates of host specificity that capture variation in parasite abundance across the phylogenetic diversity of hosts are potentially informative for species delimitation. As host specificity can act as an ecological filter to determine whether parasites have the potential to interact and reproduce with one another, the finding that closely related parasites infect mutually exclusive host groups can provide evidence for the existence of reproductively isolated evolutionary lineages.

The blood parasites in the order Haemosporida, often referred to as the ‘malaria parasites’ are a globally abundant lineage of vertebrate parasites. At least four genera of haemosporidian - *Haemoproteus*, *Leucocytozoon*, *Parahaemoproteus*, and *Plasmodium* –are common and diverse in birds [19, 20], though within these genera species limits are notoriously complex and poorly understood [13, 21, 22]. The avian malaria parasites have become an emerging model system to study the ecology and evolution of host-parasite interactions [23–25], yet their utility as a study system has been severely limited by a lack of consensus regarding species limits within the four genera. The ability to delimit species of avian malaria parasite is fundamental to inferences regarding ecological processes, such as community assembly [26, 27], as well as macroevolutionary processes such as diversification dynamics (e.g. host-switching vs. cospeciation; [28, 29]).

Two competing and highly divergent paradigms have dominated our understanding of species limits within the avian haemosporidians. Traditionally, several hundred avian haemosporidian species were described using morphology as observed under the light microscope, with host species used as a character to distinguish morphologically similar parasites under an assumption of strict host specificity [30, 31]. The rise of genetic research on avian malaria parasites has challenged the morphological species (morphospecies) concept, as the development and wide adoption of cytochrome *b* (*cytb*) as a DNA barcoding locus has revealed extreme mitochondrial diversity that is suggestive of the existence of numerous cryptic species [13, 23, 32]. The focus on using mtDNA barcodes has been driven in large part by previous studies that have found that even minute differences between haemosporidian *cytb* sequences can be used to delimit evolutionarily independent lineages, as haemosporidians have been shown to exhibit slow rates of mitochondrial evolution [33, 34]. For example, the highly influential study by Bensch et al. [13] found that in some instances a single base pair difference could be used to delimit species within the genus *Haemoproteus* (*Parahaemoproteus*), which has since been adopted as a general rule in many studies for delimiting putative haemosporidian species (e.g. [35–37]).

Using additional data types and models of species delimitation as an alternative to the morphospecies and barcode-based haemosporidian delimitation hypotheses has the potential to vastly improve our understanding of haemosporidian species limits. In particular, methods that use multi-locus sequence data and implement the multi-species coalescent model [1, 38] offer the promise of a statistical framework with which to identify reproductively isolated lineages. Coalescent methods are capable of identifying and assigning probabilities to lineages that show evidence of reproductive isolation; when applied to lineages that occur sympatrically and vary in some readily quantified ecological trait (e.g. host specificity), it is possible to capture a more nuanced view of species divergence than coarse morphology or DNA barcodes provide.

The avian malaria genus *Leucocytozoon* is of particular interest for studies of haemosporidian species limits, as species richness estimates for this genus differ dramatically between the morphospecies and mtDNA barcode hypotheses. Three morphospecies, *Leucocytozoon fringillinarum*, *L. majoris*, and *L. dubreuili* are abundant and widespread parasites of songbird (order Passeriformes) hosts in North America [31, 39]. In contrast, *cytb* surveys in North America have revealed genetically diverse *Leucocytozoon* communities infecting single host species or found in single sampling sites (e.g. 47 haplotypes [40], 19 haplotypes [41], 12 haplotypes [42]). Applying the morphospecies versus mtDNA barcode delimitations suggest vastly different estimates for *Leucocytozoon* species diversity, yet no study has tested species limits in this genus using additional data types.

Here, we use an integrative approach combining multi-locus nuclear sequence data, coalescent models of species delimitation, ecological host infection data, and morphology to conduct the first test of species limits across an entire avian haemosporidian assemblage and the first test of species limits within *Leucocytozoon*. We find that both traditional morphospecies delimitations and DNA barcodes fail to accurately capture species limits in this system, with model-based delimitation methods recovering support for at least 21 *Leucocytozoon* species across the study area. Analysis of host specificity shows that putative species are generally differentiated in their patterns of host infection, suggesting that statistical patterns of host associations should be considered an important component of integrative taxonomy of microbial symbionts in future research.

## Methods

### Sample collection

**Fig. 1:**
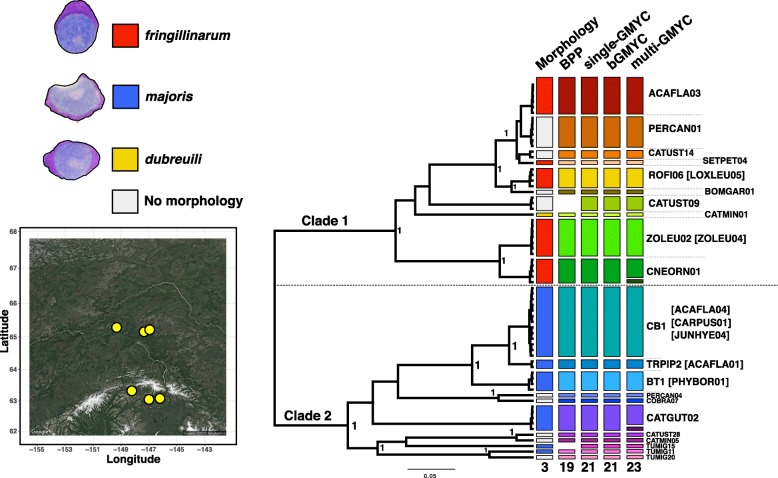
Summary of species delimitation results. Shown is a seven-gene phylogeny estimated in BEAST using the partitioned nuclear sequence, a strict molecular clock, and coalescent prior. To the left of the phylogeny are images of the three morphospecies found in this study and a map of the sites where all samples were collected generated using the *ggmap* package in R with the “get_googlemap()” function. Columns to the right of the phylogeny are the morphospecies for each sample (where available), and the results of four species delimitation methods: the A11 algorithm of BPP, single-threshold GMYC, bGMYC, and multiple-threshold GMYC. If multiple samples were supported by a species delimitation method to represent the same species, that putative species is represented in a unique color in a rectangle separated from other species. Haplotype names for each sample (or group of samples) are shown; where species delimitation methods recovered multiple haplotypes as the same species, the most common haplotype of the group is given first. Note that haplotypes CATUST09 and TUMIG15 were not included in the BPP analyses and so are not depicted in the column for BPP

We isolated *Leucocytozoon* parasites from 381 songbird (order Passeriformes) host tissues (blood and liver) from six sites in central Alaska (Fig. 1, Additional file 1: Table S1) that are accessioned in the Ambrose Monell Cryo Collection (AMCC) at the American Museum of Natural History (AMNH; Additional file 1: Table S1). All host individuals from which tissues were sampled were collected and prepared as vouchered study specimens at the AMNH following humane euthanasia according to standard practices for the collection of wild birds [43] and following approval from the Institutional Animal Care and Use Committee of the AMNH and permitted by State of Alaska Department of Fish and Game Scientific (permits 16–013 and 17–092). Blood smears were prepared at the time of collection for a subset of samples that were used for morphological study of *Leucocytozoon*. All samples were stored at − 20 C in RNALater until DNA extraction using Qiagen DNeasy Blood and Tissue kits.

### Molecular methods

Cytochrome *b* (*cytb*) barcode sequences have become the standard marker to characterize avian haemosporidian diversity [23], and so we used *Leucocytozoon cytb* haplotypes as the unit of study for tests of species limits using multi-locus sequence data (detailed below). We used the established haemosporidian *cytb* barcode primers (NFI/NR3 and FL/R2L [23, 44]) to screen all sampled hosts for *Leucocytozoon* and sequence positive infections. Infections with multiple haemosporidian *cytb* haplotypes can be common in avian communities [40, 45], and so we used a strict multi-step approach to identify host tissue samples that contained a single *Leucocytozoon* mitochondrial haplotype to be used for downstream species delimitation analyses (outlined in detail in Additional file 1: Supplementary Methods). Briefly, we used a PCR protocol that was modified from [44] by decreasing the annealing temperature and extending the annealing time to increase the chance of amplifying all mtDNA sequences present within a sample. Amplified products were directly sequenced on an ABI 3730 machine and the resulting chromatograms were assessed for signatures of overlapping sequence peaks indicative of multiple parasite haplotypes within the sample. Any samples that showed clear signatures of containing multiple *cytb* haplotypes were removed, and any samples for which the signal was unclear were re-sequenced and removed if evidence of multiple sequence peaks remained. Only sequences that exhibited a single chromatogram trace across all 479 base pairs with strong signal were analyzed further with multi-locus data. All sequences were edited using Geneious v8.0.5. Based on standard practice, all *cytb* haplotypes were assigned a unique name that was used for downstream analyses. If the *cytb* haplotype had been previously recorded in the MalAvi database [23], we used the previously assigned name for consistency. If the haplotype was new, we followed standards in the field and assigned it a new name based on the first host it was found within (e.g. the first *Leucocytozoon* haplotype from the host *Acanthis flammea* was named ACAFLA01).

We amplified partial sequences of seven nuclear protein-coding genes (*ATPase2*, *RPB1*, *POLD1*, *KPNB1*, *PRPF6*, *SEC24a*, *SF3B1*; Additional file 1: Table S2) for each single-infection sample using *Leucocytozoon*-specific nested primer pairs that were designed based on sequence data from [20, 46]. We amplified and sequenced all loci following [20], incorporating 5′ tags (CAG and M13R) in the second round of amplification for sequencing as described above. We examined nuclear sequences for overlapping peaks on sequence chromatograms as we did above for *cytb* sequences to detect any mixed infections that may have been missed by *cytb* sequencing due to PCR amplification bias. However, we did not universally discard all nuclear sequences that exhibited overlapping chromatogram peaks. We retained samples that exhibited a proportion of polymorphic sites across nuclear genes that was comparable with previously published estimates of pairwise nucleotide diversity between isolates within malaria parasite populations. This was done to distinguish nuclear variation occurring among multiple genetic clones of the same mtDNA haplotype, which are often found within infected host individuals [47, 48], from nuclear variation between putatively different species (see Additional file 1: Supplementary Methods for details). Lastly, we conducted BLAST searches on all nuclear sequences to identify and remove accidental amplifications of the avian malaria genera *Parahaemoproteus* or *Plasmodium*. In total, 69 samples exhibited consistent signatures of containing a single *Leucocytozoon* lineage across all eight loci (*cytb* and seven nuclear loci). These samples were retained for species delimitation analyses.

### Morphology

We scanned all available blood smears from single infection samples for *Leucocytozoon* at 200X across 50 fields and recorded the morphotype of all *Leucocytozoon* parasites that were observed. Following the descriptions in [31], we classified each parasite as belonging to the *L. fringillinarum*, *L. majoris*, or *L. dubreuili* morphospecies (no other morphospecies were observed).

### Phylogenetic analyses

We estimated Bayesian and maximum likelihood phylogenies for all single-infection *Leucocytozoon* samples using the full nuclear (seven gene) dataset implemented in BEAST v1.8 [49]. We used jModelTest 2 [50] to estimate the best-fit substitution model for each locus, and implemented a strict molecular clock and coalescent tree prior for 30 million generations, discarding the first 10% as burn-in. We used Tracer v1.6 [51] to ensure all parameters reached sufficient ESS values. We also estimated a maximum likelihood phylogeny using RAxML 8.2.3 [52], implementing the GTRGAMMA substitution model and rapid bootstrapping. All analyses used the *Parahaemoproteus cytb* haplotype SPIARB01 as the outgroup. As the use of the outgroup introduced gaps in several alignments (particularly in *RPB1*), we used the program Gblocks [53] to eliminate gaps and poorly aligned regions prior to analysis. Haplotype networks for individual nuclear loci were visualized using the TCS algorithm [54] implemented in PopART [55].

### Species delimitation analyses

We used two species delimitation approaches to test species limits among the sampled *cytb* haplotypes, as each method implements different assumptions about the speciation process and thus we sought to test for consensus across different methodologies [56]. First, we used the single- and multiple-threshold generalized mixed Yule coalescent model (GMYC; [6, 57]) using the R package *splits* [58]. The GMYC model integrates the Yule and coalescent models to identify the branching pattern threshold point between speciation (Yule) and population genetic (coalescent) processes, and is ideal for our dataset that is unbalanced with respect to sampling within and between different *Leucocytozoon cytb* haplotypes (e.g. some haplotypes were sampled multiple times, while others are represented as singletons; [59]). We also used the Bayesian implementation of the GMYC model, bGMYC [60], which accounts for uncertainty in branch lengths and tree topology by sampling from the posterior distribution of trees. We conducted eight separate GMYC analyses: one analysis using trees estimated for each nuclear locus, as well as an analysis using a tree estimated from a concatenated analysis of all seven nuclear loci. For single-locus analyses we generated ultrametric gene trees as input for GMYC analysis using BEAST v1.8, implementing identical parameters as described for the concatenated analysis and as suggested by [59]. The analysis using the tree estimated from the concatenated alignment was performed due to varying proportions of missing data in each individual nuclear gene alignment; although the GMYC model was designed as a species delimitation tool for single-locus barcode datasets, there is no inherent limitation to the model that precludes it from being used on concatenated datasets [58]. The outgroup and identical sequences were removed prior to all GMYC analyses. For bGMYC analyses, we ran MCMC chains for 50,000 generations per tree on 100 topologies from the posterior distribution of the BEAST analysis, discarding the first 50% of steps as burn-in for each tree with a thinning of 100.

We also conducted joint species delimitation and species tree estimation using the A11 algorithm in the program BPP v3.3 [61]. This program uses a Bayesian framework and the multispecies coalescent model to account for gene tree-species tree conflict due to incomplete lineage sorting [62–64]. We conducted BPP analyses separately on two *Leucocytozoon* clades to avoid problems caused by model misspecification among distantly related lineages. Pairwise *cytb* divergence was an average of 4.1% and 3.9% within the two clades, respectively, while average divergence between clades was 8%. Two divergent *cytb* haplotypes, CATUST09 and TUMIG15, differed by at least 5.8% and 6.8%, respectively, from all other haplotypes and so were not included in BPP analyses to avoid model misspecification. As a primary goal of this study was to test whether all haemosporidian *cytb* haplotypes represent reproductively isolated lineages, we set all *cytb* haplotypes as unique populations in BPP analyses, as BPP will attempt to lump populations into species but will not split populations [61]. We retained all singleton *cytb* haplotypes for these analyses (i.e. haplotypes that were represented by one sample), as BPP has been shown to be robust to the inclusion of species represented by a single individual [65]. Following standard approaches [16, 66] we conducted four BPP analyses using different combinations of diffuse population size (θ) and divergence time (τ) priors to evaluate the sensitivity of analyses to variation in these parameters. All priors were set as gamma distributions (α, β) with mean α/β and variance α/β^2^. We conducted analyses using (α, β): 1) large population size (1, 10) and large divergence time (1, 10); 2) large population size (1, 10) and small divergence time (1, 1000); 3) small population size (1, 1000) and large divergence time (1, 10); and 4) small population size (1, 1000) and small divergence time (1, 1000). Each analysis was run twice using different seeds to test for variation among runs. We ran each analysis for 250,000 generations, sampling every 5 generations and using a burn-in of 10% All analyses were run using the Gblocks alignments and with the option cleandata = 0.

### Host specificity analyses

We sought to use host specificity as an independent data source to test whether *Leucocytozoon cytb* haplotypes that were identified as reproductively isolated lineages differed in their infection patterns across the host community, as we hypothesized that species-level divergences are associated with ecological divergence in patterns of host use. Though host specificity would ideally be integrated directly with parasite genetic data for species delimitation as has been done previously with morphological data [67], host specificity is not measured as a characteristic of individual parasites (rather it is measured as a characteristic of parasite populations). An additional challenge is that while several commonly used metrics for parasite host specificity are capable of quantifying the relative host breadth of a parasite [68, 69], these metrics are not capable of capturing relative differences in the composition of the host community that a parasite infects. For instance, traditional metrics can determine whether two parasite species are host specialists, meaning that they infect a specific phylogenetic subset of the host community, though these methods cannot capture whether the two parasite species are specialized on the same host group or different host groups. To characterize pairwise differences in host specificity among *Leucocytozoon cytb* haplotypes that also capture differences in infection patterns across the host phylogeny, we calculated the pairwise weighted UniFrac distance [70] using the R package *phyloseq* [71]. Weighted UniFrac uses a phylogeny as well as species abundances to estimate distances among biological communities. Here, we treated the hosts that are infected by each *Leucocytozoon cytb* haplotype as “communities” to quantify differences in *Leucocytozoon* infection patterns across phylogenetic host space. We used the weighted UniFrac distance to account for differences in abundance of parasites across host species, so that rare ‘spillover’ infections into a host in which a parasite cannot complete its lifecycle have less influence than hosts that are frequently infected and ecologically important for the parasite. When calculating pairwise UniFrac distances we considered all detections of each *Leucocytozoon cytb* haplotype, including those that were found in mixed infections, and the abundance of a host within a parasite’s “host community” was simply the total number of times that host was found to be infected by a given *Leucocytozoon cytb* haplotype across all 381 host samples. For the host phylogeny we downloaded a distribution of 100 bird phylogenies from www.birdtree.org using the “Ericson all species” option, and used TreeAnnotator v1.8 [49] to produce a single maximum clade credibility tree. We summarized differences in weighted UniFrac distances among *cytb* haplotypes using principal coordinate analysis implemented in the R package *ape* [72]. To test whether closely related *cytb* haplotype pairs (< 2.5% sequence divergence) have more divergent weighted UniFrac distances than expected by chance, we randomized the host-parasite matrix using the “permatfull” function in the R package *vegan* [73], preserving both column and row totals (fixedmar = “both”). We randomized the matrix 1000 times to generate a null distribution of host-parasite occurrences, and tested whether the observed UniFrac distance between any chosen *cytb* haplotype pair was significantly higher or lower than expected by chance (*p* < 0.05 threshold). UniFrac distances were not calculated for *Leucocytozoon cytb* haplotypes that were only detected once due to insufficient data.

## Results

We screened 381 songbird hosts from central Alaska for *Leucocytozoon*, from which we isolated 69 single-infection samples representing 28 *Leucocytozoon cytb* haplotypes. These 28 *cytb* haplotypes served as the basis for further analyses that tested: 1) whether different *cytb* haplotypes shared nuclear alleles; 2) whether different *cytb* haplotypes were supported as conspecific or heterospecific using coalescent species delimitation analyses, and 3) whether different *cytb* haplotypes exhibited significantly different host infection patterns. Of these 28 *Leucocytozoon cytb* haplotypes, 18 haplotypes were represented by a single sample for species delimitation analyses while the remaining haplotypes were represented by two to ten samples each (Additional file 1: Table S3).

**Fig. 2:**
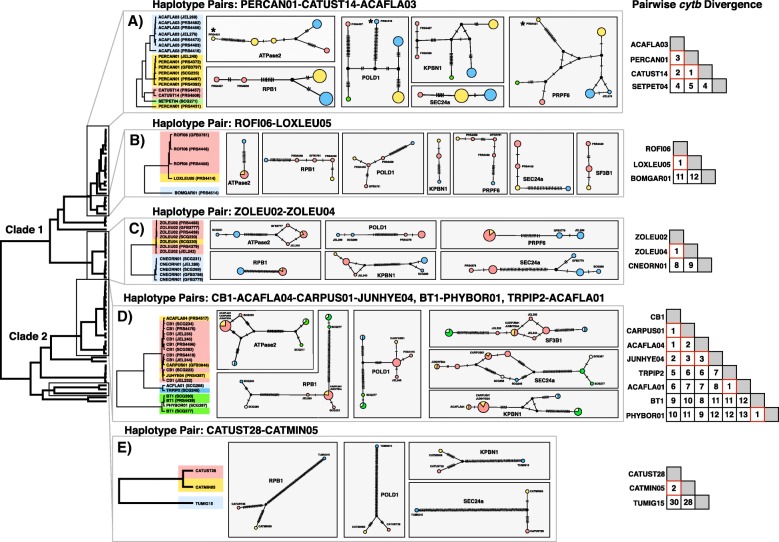
Nuclear divergence of *Leucocytozoon cytb* haplotypes. Depicted are five clades that contain pairs of *cytb* haplotypes that differ by one to three base pairs (as shown in the distance matrices of *cytb* differences to the right of the networks). In each clade haplotypes are given a different color that corresponds to the haplotype networks. **a** haplotypes PERCAN01, ACAFLA03, and CATUST14, which differ by one to three bases, are differentiated across all nuclear loci and never share nuclear alleles. The three sequences that are marked by asterisks represent outlier samples of high divergence that were removed prior to species delimitation analysis due to the possibility that they contained mixed infections; **b**) ROFI06 and LOXLEU05 differ by a single base pair and are weakly differentiated across nuclear loci, sharing nuclear alleles for a single locus; **c**) ZOLEU02 and ZOLEU04 differ by a single base pair and share alleles across multiple nuclear loci; **d**) haplotypes CB1, JUNHYE04, CARPUS01, and ACAFLA04 differ by one to three bases, but share alleles across multiple nuclear loci. In addition, haplotype pairs TRPIP2/ACAFLA01 and BT1/PHYBOR01 differ by a single base pair but share nuclear alleles; **e**) CATUST28 and CATMIN05 differ by two bases but are highly differentiated across all nuclear loci. Note that not all nuclear loci are depicted for each clade due to insufficient sample sizes for some haplotypes

Across all 381 sampled hosts we recorded the 28 target *cytb* haplotypes a total of 406 times (including both single infections and mixed infections; Additional file 1: Table S3). The 69 samples that were retained for species delimitation were isolated from 21 host species across six sites (Additional file 1: Table S1). Nuclear loci amplified and sequenced with varying success, ranging from 66 (*KPNB1*) to 39 (*SF3B1*) samples sequenced per locus (out of 69 total samples; Table 1, see Additional file 1: Table S4 for GenBank accession numbers). We discovered that two samples (PRS4416 haplotype ACAFLA03 and PRS4431 haplotype PERCAN01) exhibited highly heterogeneous divergence from other samples of the same *cytb* haplotype across nuclear loci, with pairwise divergences among loci varying from 0% to as high as 9.3% (Fig. 2, Additional file 1: Tables S5–6). This signature of high genetic divergence from other samples of the same *cytb* haplotype at some, but not all, loci suggests that PRS4416 and PRS4431 contained mixed infections that were not seen on sequence chromatograms and so the two samples were removed from further analysis.

**Table 1:** Nuclear genes sequenced for multi-locus species delimitation in this study

Gene	Aligned length (bp)	Aligned length (bp) no outgroup	*N*	Model
ATPase2	517	452	63	GTR + I + G
RPB1	1035	854	65	GTR + I + G
POLD1	484	484	54	GTR + I + G
KPBN1	430	430	66	GTR + I + G
PRPF6	530	455	41	HKY + G
SEC24A	628	610	58	GTR + G
SF3B1	388	388	39	HKY + G

Alignment lengths, number of samples that amplified and sequenced successfully, and best fit model of nucleotide substitution as estimated in jModelTest2 for each gene. Alignment lengths differ in some loci with the outgroup removed due to the deletion of gaps

### Species delimitation analyses

**Fig. 3:**
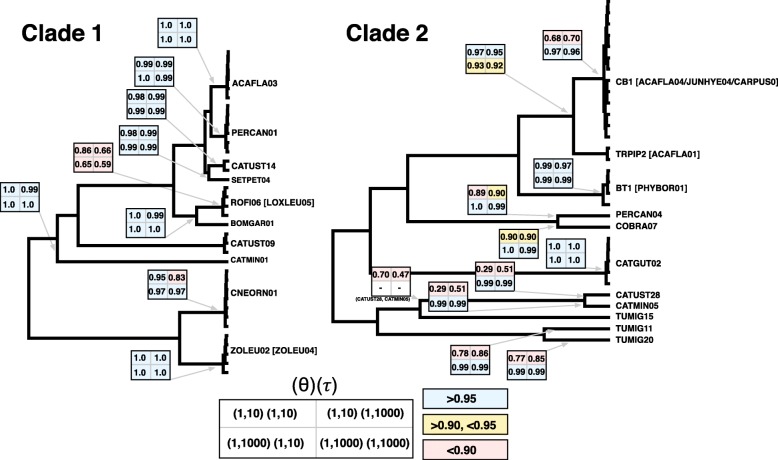
Results of BPP species delimitation using the A11 algorithm. Boxes at each node depict the results of the four analyses that were conducted using different diffuse priors for population size and divergence time. If a node was supported to delimit a species with posterior probability > 0.95, the square for that analysis is depicted in blue (squares are depicted in yellow and red for posterior probabilities less than 0.95 and 0.90, respectively). Note that no posterior probabilities are depicted for lineages CATUST09 and TUMIG15, as they were removed from this analysis due to high divergence from all other haplotypes

Species delimitation analyses produced estimates that varied from 19 species (BPP) to 23 species (mGMYC), with sGMYC and bGMYC analyses both recovering support for 21 species (Fig. 1, Additional file 1: Table S7). BPP recovered consistent support for 19 species using different priors (Fig. 3), though note that this analysis excluded haplotypes CATUST09 and TUMIG15, which were recovered as reproductively isolated lineages in all GMYC analyses. Posterior probabilities for BPP delimitations were consistently above 0.95 (17 of 19 delimited species) using a prior that reflects small population size (θ = 1, 1000). Just one putative species consisting of haplotypes ROFI06 and LOXLEU05 was not strongly supported for any combination of BPP priors (posterior probability 0.59–0.86; Fig. 3), though these haplotypes were supported as conspecific in all GMYC analyses. Across the seven nuclear markers that we sequenced for this study, single-threshold GMYC species delimitation estimates varied widely for individual loci (range: 12–21 species, Additional file 1: Table S8), and only a single locus obtained the same number of estimated species as the concatenated dataset (*KPBN1*, 21 species). Using microscopy we were able to assign 16 of the 28 identified *cytb* haplotypes to morphological groups (Additional file 2: Figure S1) that matched the previously described morphospecies *Leucocytozoon fringillinarum*, *L. majoris*, and *L. dubreuili*. Samples with *L. fringillinarum* and *L. majoris* morphologies each composed large clades that contained multiple putative species that were identified in species delimitation analyses. The *L. dubreuili* morphotype was found only for *cytb* haplotype CATMIN01, which was nested within the *fringillinarum* clade.

### Association between cytb and nuclear sequence divergence

We focused on the association between mtDNA and nuclear genetic divergence among closely related *cytb* haplotypes, given previous support for the finding that a single base pair difference (0.2%) between *cytb* barcodes can signal reproductive isolation among avian haemosporidian lineages [13, 22]. We recovered multi-locus sequence data for 14 pairs of *cytb* haplotypes (17 total haplotypes) that differed by 0.2–0.6% sequence divergence (one to three base pairs; seven haplotype pairs differed by one base pair, four haplotype pairs differed by two base pairs, and three haplotype pairs differed by three base pairs; Fig. 2, Table 2). We found that in six of seven instances, *cytb* haplotypes that differed by one base pair were recovered as the same species in all species delimitation analyses and shared alleles for at least one nuclear locus (Table 2). However, one pair of lineages (PERCAN01 and CATUST14) that differed by a single *cytb* base pair were recovered as different species in all analyses. Furthermore, PERCAN01 and CATUST14 were divergent across all nuclear loci (1.1 to 5.2% pairwise divergence across loci), and never shared nuclear alleles.

**Table 2:** Summary of genetic and ecological divergence among closely related *cytb* haplotype pairs

*Cytb* Haplotype Pair	*Cytb* Divergence	Share nuclear alleles [N loci]	Nuclear Divergence (%) [Min-Max]	N Analyses delimited as species	UniFrac significant
ZOLEU02/ZOLEU04	1 bp (0.2%)	Yes [3]	0–0.27	0/4	–
CB1/CARPUS01	1 bp (0.2%)	Yes [5]	0.04–0.66	0/4	–
CB1/ACAFLA04	1 bp (0.2%)	Yes [2]	0.23–0.66	0/4	–
BT1/PHYBOR01	1 bp (0.2%)	Yes [5]	0–1.01	0/4	–
TRPIP2/ACAFLA01	1 bp (0.2%	Yes [4]	0–0.66	0/4	–
ROFI06/LOXLEU05	1 bp (0.2%)	Yes [1]	0–3.17	0/4	Yes (lower)
PERCAN01/CATUST14	1 bp (0.2%)	No	1.07–5.19	4/4	Yes (higher)
CB1/JUNHYE04	2 bp (0.4%)	Yes [5]	0.03–0.50	0/4	–
CARPUS01/ACAFLA04	2 bp (0.4%)	Yes [1]	0.48–0.63	0/4	–
CATUST28/CATMIN05	2 bp (0.4%)	No	1.66–9.51	4/4	No
ACAFLA03/CATUST14	2 bp (0.4%)	No	1.42–4.62	4/4	Yes (higher)
CARPUS01/JUNHYE04	3 bp (0.6%)	Yes [4]	0–0.71	0/4	–
ACAFLA04/JUNHYE04	3 bp (0.6%)	Yes [1]	0–0.70	0/4	–
ACAFLA03/PERCAN01	3 bp (0.6%)	No	0.36–4.38	4/4	Yes (higher)
TRPIP2/CB1	5 bp (1%)	No	2.31–7.55	4/4	Yes (higher)
ZOLEU02/CNEORN01	8 bp (1.6%)	No	0.31–8.72	4/4	Yes (higher)
CB1/BT1	9 bp (1.8%)	No	5.74–14.39	4/4	Yes (higher)
TRPIP2/BT1	11 bp (2.2%)	No	5.9–14.7	4/4	Yes (higher)

**Fig. 4:**
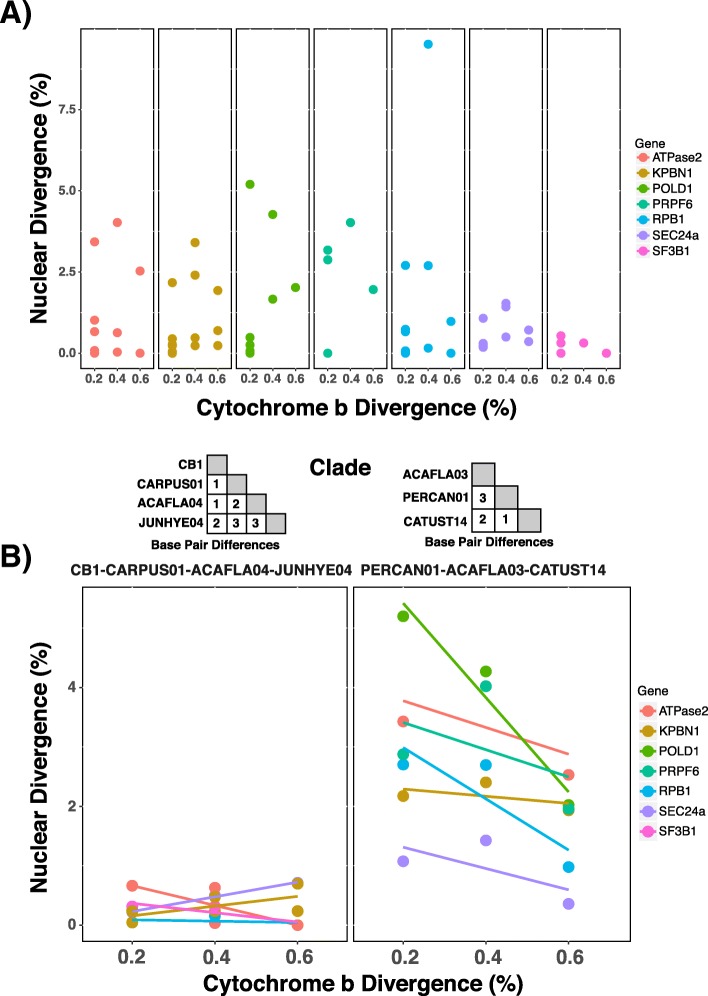
The relationship between *cytb* and nuclear divergence in *Leucocytozoon* is inconsistent and clade-dependent. **a** Among 14 pairs of *Leucocytozoon cytb* haplotypes (17 haplotypes total), the divergence between them is not strongly associated with nuclear divergence for the seven loci sequenced in this study. **b** The relationship between *cytb* divergence and nuclear divergence appears to be clade dependent, as the clade consisting of CB1/ACAFLA04/CARPUS01/JUNHYE04 (which were all found to be conspecific) exhibits no association between *cytb* divergence and nuclear divergence while the clade consisting of ACAFLA03/CATUST14/PERCAN01 exhibits strong nuclear differentiation across the same scale of *cytb* divergence, though nuclear divergence does not appear to increase with *cytb* divergence

Shown for each *cytb* haplotype pair: the number of base pair differences between haplotypes, whether the haplotypes were found to share alleles for at least one nuclear locus, the observed range of pairwise genetic divergence for nuclear loci between the two haplotypes, the number of species delimitation analyses for which the pair was delimited as separate species, and whether the weighted UniFrac distance between them was significantly different (lower or higher than expected by chance; this metric was not able to be calculated for all comparisons due to sample size of one of the haplotypes). The 14 haplotype pairs that are 02–0.6% divergent are depicted in Fig. 4a

Species delimitation and nuclear divergence were inconsistent among *cytb* haplotypes that differed by two to three base pairs, as some haplotype pairs were recovered as conspecific and shared nuclear alleles while others were delimited as different species and had divergent nuclear sequences (Table 2). There appeared to be strong differences in the association between *cytb* and nuclear genetic divergence among clades, particularly between the clade composed of CB1, CARPUS01, JUNHYE04, and ACAFLA04 which showed virtually no nuclear divergence among *cytb* haplotypes that differed by as many as three base pairs and the clade containing the *cytb* haplotypes ACAFLA03, CATUST14, and PERCAN01 which exhibited as much divergence between *cytb* haplotypes that differ by one base pair as those that differ by three base pairs (Fig. 4).

### Host specificity analyses

**Fig. 5:**
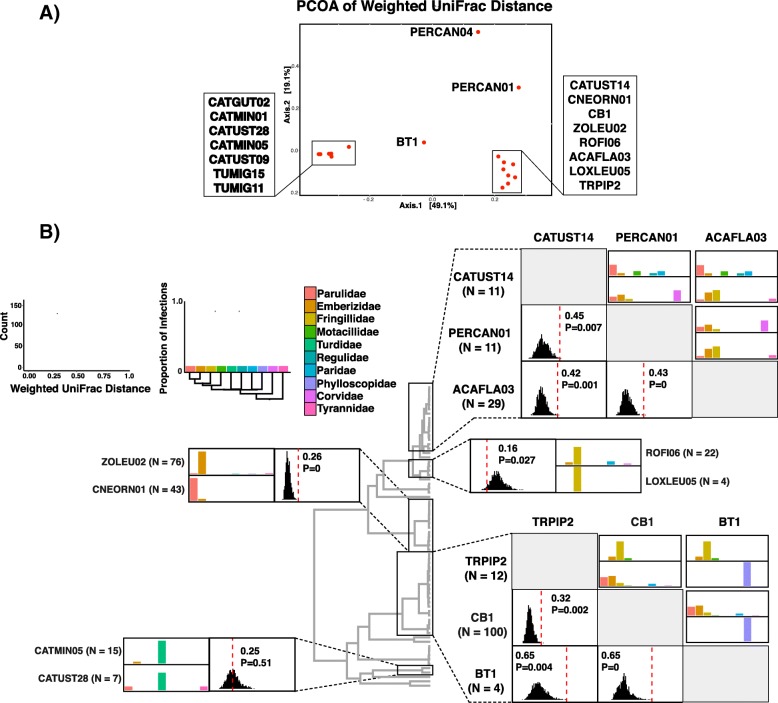
Patterns of putative *Leucocytozoon* species infection across host species. **a** Principal coordinates analysis of weighted UniFrac distances among 18 *Leucocytozoon cytb* haplotypes that were sampled at least twice across 381 hosts. **b** Paired histograms of randomized weighted UniFrac distance and barplots demonstrating differential infection across potential host families for closely related *cytb* haplotype pairs. Vertical lines in histograms depict the observed UniFrac distance for that putative species pair, which is also shown above each histogram with its associated significance value. Colors of the barplots correspond to the host phylogeny shown in the key to the left. UniFrac distances and barplots are depicted in two pairwise matrices for clades that contain three putative species; UniFrac distance is shown below the diagonal for each cytb haplotype pair, while above the diagonal are host infection barplots with the haplotype along the lower diagonal representing the top barplot

We used the weighted UniFrac metric to characterize how putative *Leucocytozoon* species infect the host community while accounting for both host phylogeny and the abundance of parasites across all host species. We restricted host specificity analyses to 18 *Leucocytozoon* cytb haplotypes that were found to infect at least two host individuals in our sample, with the number of infections per *Leucocytozoon cytb* haplotype ranging from 2 to 101 host-parasite observations (median 11 infections per *cytb* haplotype; Additional file 1: Table S3). We found that closely related (< 2.5% sequence divergence) *cytb* haplotypes that had been supported by species delimitation analyses were generally strongly differentiated in their patterns of host use (Fig. 5). *Leucocytozoon cytb* haplotypes were largely separated by specificity to host family: 14 *cytb* haplotypes were found predominantly (> 70% of infections) in one host family, while four *cytb* haplotypes exhibited generalist strategies where no host family reached 50% of total infections. To test whether *cytb* haplotypes within the same clade exhibited significantly different patterns of host infection, we conducted pairwise randomization tests of weighted UniFrac distance. We found that the observed UniFrac distance was significantly higher (i.e. host utilization was more divergent) than the randomized distribution (Table 2) in all but two instances. One exception was for the *cytb* haplotypes CATUST28 and CATMIN05, which were supported as different species in all species delimitation analyses, but were not significantly differentiated in their host infection patterns (*p* = 0.513). An additional randomization test found that the *cytb* haplotypes ROFI06 and LOXLEU05 were more similar in host specificity than expected by chance (*p* = 0.027), though these haplotypes were supported as conspecific in species delimitation analyses.

## Discussion

Morphologically ambiguous complexes of microbial symbionts pose difficult challenges for the identification of species boundaries, though an integrative approach combining coalescent species delimitation methods and ecological data can provide important insights into the diversity of symbiont communities and the complexity of host-symbiont interactions. Here, we applied a suite of coalescent species delimitation methods and analyses of host specialization to test for species limits within an assemblage of morphologically conserved but genetically diverse avian malaria parasites in the genus *Leucocytozoon*. We found that three abundant morphospecies in central Alaska likely consist of at least 21 reproductively isolated species that vary widely in their interactions with the host community.

### Cryptic morphology has masked a diverse *Leucocytozoon* assemblage

Our analysis provides strong evidence that neither traditional morphospecies concepts nor mtDNA barcodes alone adequately characterize species diversity in *Leucocytozoon*. First, we show definitively that the morphospecies “*L. fringillinarum”* and “*L. majoris”* are not valid species, but rather that each represent a complex of cryptic species with divergent host infection patterns. Previous analyses using mtDNA have found evidence that *L. fringillinarum* and *L. majoris* are not monophyletic [74, 75], though this is the first study to rigorously test for species delimitations in this genus using multi-locus data. Morphology did exhibit strong phylogenetic signal, as the *fringillinarum* and *majoris* morphotypes each formed clades in which we delimited numerous putative species. Though the *dubreuili* morphotype was found for a single *cytb* haplotype in this study, we anticipate that further sampling across the global distribution of this morphotype will also reveal extensive cryptic species-level diversity.

While the evidence from coalescent analyses suggest that there are at least 21 reproductively isolated lineages of *Leucocytozoon* in central Alaska, it is important to note that the species we delimit here are at this point still putative. One analysis (mGMYC) recovered support for as many as 23 species and in two cases delimited multiple species within a single *cytb* haplotype; however, this method tends to over-split species [58, 76]. We suggest that there is strong evidence for 21 species in this assemblage when combining congruence across species delimitation analyses and ecological host specificity data (discussed in detail below). Additional studies that examine the potential for these putative species to sexually reproduce are warranted [77], though the observation that they occur sympatrically across the study region and yet appear to have ceased gene flow and in most cases have evolved divergent ecologies suggests that we have delimited reproductively isolated units.

### Putative *Leucocytozoon* species exhibit a range of genetic and ecological divergence despite shallow mitochondrial differentiation

While the morphospecies concept that was traditionally used to delimit *Leucocytozoon* species clearly underestimates *Leucocytozoon* species diversity, our results indicate that the mtDNA barcoding approach is likely to overestimate species diversity. In particular, a “one base pair rule” whereby a single base pair difference in the *cytb* barcoding fragment indicates reproductive isolation has become a popular means with which to delimit species in macroecological studies of haemosporidian diversity [35, 36, 78] despite mixed support for the rule [22, 79, 80]. We recovered support for 21 species of *Leucocytozoon* despite studying 28 *cytb* haplotypes, several of which differed by a single base pair.

Across 14 pairs of *cytb* haplotypes that differed by one to three base pairs (0.2–0.6%), we observed no consistent relationship between *cytb* and nuclear divergence or the results of species delimitation analyses. *Cytb* haplotype pairs that differed by a single base tended to be recovered as conspecific and share nuclear alleles (six of seven instances), with one notable exception between the *cytb* haplotypes CATUST14 and PERCAN01. Among *cytb* haplotype pairs that differed by two or three bases, there was no pattern between nuclear divergence and species delimitation: four haplotype pairs were found to be conspecific with little to no nuclear divergence, while three were found to be heterospecific and were divergent across nuclear loci. Interestingly, there was a strong effect of phylogeny – similar scales of *cytb* divergence reflected very different nuclear divergences in two well-sampled clades. One possible explanation for this pattern is variation in effective population size caused by population bottlenecks or differences in life histories [81], as larger populations are able to retain more genetic variation (e.g. *cytb* polymorphisms may be retained in species with large effective population sizes and lost in those with small population sizes). Overall, these results indicate that there is no consistent “barcode gap” [82] among *Leucocytozoon cytb* haplotypes, as shallow mitochondrial divergence can reflect divergent histories of reproductive isolation in different lineages.

Species delimitations were supported by an analysis of how these putative species infect the host community based on sequencing of 406 *Leucocytozoon* infections from 381 sampled hosts. Generally, the putative species identified by genetic species delimitation infected significantly different proportions of the host community than their closest relatives based on analysis of the weighted UniFrac metric. However, similar to the genetic results we observed a range of differentiation in host specificity among putative species with closely related species pairs exhibiting divergent (e.g. ACAFLA03/CATUST14) or nearly identical (e.g. CATUST28/CATMIN05) host infection patterns. It is clear from this analysis that the evolution of host specificity in this system is dynamic and can be informative for species delimitation, especially when comparing putative species that are weakly diverged across barcode loci. As host specificity metrics can only be used to characterize parasite populations, and not individual parasites, simultaneous analysis of host specificity and genetic data within a single analytical framework is not possible. In systems that are amenable to experimentation, it may be possible to measure the ability of individual parasites to infect different host groups, though such an analysis is likely not realistic for most host-parasite systems. Future research on this system will also benefit greatly from studies of how these putative species differ in their patterns of vector (i.e. invertebrate host) use. It is possible that these putative species differ in the species of blackfly vector that they are transmitted by, potentially providing additional evidence for reproductive isolation [83–85]. However, research on *Leucocytozoon* infection patterns in blackflies in Colorado [86] found that a single blackfly species can vector a broad evolutionary diversity of *Leucocytozoon* haplotypes. For instance, the blackfly *Simulium silvestre* was found to harbor 32 divergent *Leucocytozoon cytb* haplotypes (including seven haplotypes found in this study), demonstrating that vectors may not play an important role as ecological filters within this host-parasite community [87].

It remains likely that the putative species identified here do not represent all *Leucocytozoon* species present in the studied region. Across all of the screened host samples we identified five additional *cytb* haplotypes that were found to infect hosts at low frequencies that we were unable to generate nuclear sequence data for (ACAFLA02, CATMIN02, TUMIG12, COLBF21, COLBF28). Furthermore, this study only examined songbird (order Passeriformes) hosts – *Leucocytozoon* is known to be abundant and diverse in Alaska in several other avian orders (e.g. Galliformes [88] and Anseriformes [89]). Further sampling will likely reveal an even more diverse and complex *Leucocytozoon* community than we report in the present study, potentially providing additional support for the hypothesis that global species-level diversity of *Leucocytozoon*, if not all avian haemosporidians, is vastly underestimated.

The finding that no universal mtDNA barcode gap exists for *Leucocytozoon* raises the question of whether there is a need for a paradigm shift in our approach to delimiting species of avian haemosporidians [21] and other groups for which classical barcoding approaches fail. Ideally, future studies that rely on species-level delimitations of avian haemosporidians will use an integrative taxonomic approach combining multi-locus sequence and ecological data (e.g. vertebrate and vector host use data). Perhaps more realistically, at least one additional line of evidence in conjunction with *cytb* barcode sequence should be sought in order to accurately delimit haemosporidian species (Additional file 1: Table S9). For instance, [90] used host-parasite interaction data to delimit evolutionary lineages of avian haemosporidians from *cytb* barcode data (though they used a different segment of *cytb* than what was sequenced in the present study). When sequencing additional markers is not possible, future research should assess the sensitivity of analyses to using alternative species delimitations for *cytb* haplotypes, and consider removing rare haplotypes that do not have sufficient sample sizes to test for divergence in the ecological trait of interest (e.g. host specificity or associations with abiotic environmental variables). Public databases such as MalAvi [23] that record both genetic and ecological data will be integral in this regard, and we encourage authors to deposit complete records (i.e. each individual host-parasite association) of parasite prevalence across host communities for maximum utility of the data.

## Conclusions

We show that both morphology and mtDNA barcodes are incapable of accurately delimiting species in the avian malaria genus *Leucocytozoon*, and that integrating multi-species coalescent models that assess evolutionary independence with host specificity data recovers support for at least 21 species occurring sympatrically in central Alaska. We find that evidence for reproductive isolation among putative *Leucocytozoon* species can occur with minimal mitochondrial divergence – in one case just a single base pair difference – but across all putative species we observed a continuum of genetic and ecological divergence that was not closely associated with differences in the popular *cytb* barcoding locus. These findings suggest that the process of divergence in avian malaria parasites is complex and variable with respect to genetic and ecological differentiation. Avian haemosporidian mitochondrial diversity continues to be uncovered at a rapid rate, though if we are to understand the ecological and evolutionary patterns and processes that influence haemosporidian diversity it will be critical that we are accurately delimiting evolutionarily independent lineages. Assessing species limits using the multi-species coalescent model and host specificity provided a more nuanced view of species divergence in this system than would have been possible with either data type alone, suggesting that a shift in species delimitation approaches for malaria parasites and other diverse microbial symbionts is warranted.

## Additional files

Additional file 1: Additional details of sampling, methodology, and results. This file contains supplementary methods, data on the hosts from which *Leucocytozoon* samples were isolated (**Table S1**), the PCR primers used to generate *Leucocytozoon* nuclear sequence data (**Table S2**), a table showing the total number of samples for each haplotype used for species delimitation analyses (**Table S3**), GenBank accession numbers for each sample (**Table S4**), pairwise genetic distance matrices among samples of the cytb haplotype PERCAN01 (**Table S5**), pairwise genetic distance matrices among samples of the cytb haplotype ACAFLA03 (**Table S6**), posterior probabilities of species delimitation for bGMYC analyses (**Table S7**), results of single-locus species delimitation analyses (**Table S8**), and an overview of the suggested framework for identifying putative species of microbial symbionts (**Table S9**). (DOCX 245 kb)

Additional file 2: Microscopic images of *Leucocytozoon cytb* haplotypes included in this study. This file contains microscopic images for each *Leucocytozoon cytb* haplotype for which material was available. (DOCX 14716 kb)

## Abbreviations

Bp: Base pairs
BPP: Bayesian Phylogenetics and Phylogeography
*Cytb*: Cytochrome *b*
GMYC: General Mixed Yule Coalescent
