# Supplementary material for: Integrating coalescent species delimitation with analysis of host specificity reveals extensive cryptic diversity despite minimal mitochondrial divergence in the malaria parasite genus Leucocytozoon
Source: BMC Evol Biol. 2018 Aug 30;18:128. doi: 10.1186/s12862-018-1242-x (PMC6117968; doi:10.1186/s12862-018-1242-x)
Supplement: Supplementary file 1 — Additional details of sampling, methodology, and results. This file contains supplementary methods, data on the hosts from which Leucocytozoon samples were isolated (Table S1), the PCR primers used to generate Leucocytozoon nuclear sequence data (Table S2), a table showing the total number of samples for each haplotype used for species delimitation analyses (Table S3), GenBank accession numbers for each sample (Table S4), pairwise genetic distance matrices among samples of the cytb haplotype PERCAN01 (Table S5), pairwise genetic distance matrices among samples of the cytb haplotype ACAFLA03 (Table S6), posterior probabilities of species delimitation for bGMYC analyses (Table S7), results of single-locus species delimitation analyses (Table S8), and an overview of the suggested framework for identifying putative species of microbial symbionts (Table S9). (DOCX 245 kb) [file 12862_2018_1242_MOESM1_ESM.docx]

**Additional File 1 for: “Integrating coalescent species delimitation with analysis of host specificity reveals extensive cryptic diversity despite minimal mitochondrial divergence in the malaria parasite genus *Leucocytozoon”***

**Supplementary Methods**

We identified single-haplotype *Leucocytozoon* samples using a multi-step approach. First, we amplified *cytb* for each sample using a PCR protocol that was modified from Hellgren et al. (2004) to increase the chance of amplifying all mtDNA sequences present within a sample. This modified PCR protocol (94 °C for 35s, 48 °C 1 min, 68 °C for 40s for 35 cycles) increased the time for denaturing and annealing and lowered the annealing temperature relative to the standard protocol. This protocol was repeated for the outer and nested reactions, and amplified products were sequenced bi-directionally on an ABI 3730 machine. Any sample that demonstrated multiple peaks in sequence chromatograms that were clearly visible in both sequencing directions was considered to be a co-infection and removed from further analysis. If a sample exhibited minor peaks that could not be readily distinguished from the noise generated during sequencing, the sample was re-amplified and re-sequenced. If the resulting sequencing reaction yielded no minor peaks we moved forward with the sample as a single infection, though any samples that exhibited the same minor peaks on sequence chromatograms after re-sequencing were removed.

Although haemosporidians are haploid in their vertebrate hosts, many naturally occurring infections contain multiple genetically unique clones of the same species (Manske et al. 2012, Hicks and Schall 2014). Evidence of ‘mixed’ bases (i.e. overlapping chromatogram peaks) in haemosporidian nuclear loci can therefore be a sign of an infection with multiple malaria parasite species or multiple genetic clones of the same mtDNA haplotype. As a guideline, if a sample exhibited polymorphism in at least one locus we retained the sample for further analysis if the proportion of polymorphic sites summed across loci was within the bounds of what has been reported from previous surveys of pairwise nucleotide diversity among isolates within malaria parasite populations (Volkman et al. 2006, *Plasmodium* *falciparum*: 1.16 x 10^-3^; Tachibana et al. 2012, *Plasmodium* *cynomolgi*: 5.41 x 10^-3^; Assefa et al. 2015, *Plasmodium* *knowlesi*: 6.03 x 10^-3^). If a sample exhibited a proportion of polymorphic sites that fell outside of the upper bound of this range, we removed the entire sample from further analysis. Among samples that we retained that did exhibit polymorphism in nuclear loci, the observed proportion of differences varied from 2.5 x 10^-4^ to 3.4 x 10^-3^. Just three samples (GFB3788-CNEORN01, JEL251-CATUST09, and PRS4389-CATUST09) exhibited a proportion of polymorphic sites greater than 2.0 x 10^-3^ (approximately eight polymorphic sites across ~4,000 bp); removing these samples had no impact on the results of downstream species delimitation analyses, and so the samples were retained.

Assefa S, Lim C, Preston MD, Duffy CW, Nair MB, Adroub SA, et al. Population genomic structure and adaptation in the zoonotic malaria parasite *Plasmodium knowlesi*. P Natl Acad Sci USA. 2015; 112:13027–32.

Tachibana S-I, Sullivan SA, Kawai S, Nakamura S, Kim HR, Goto N, et al. *Plasmodium cynomolgi* genome sequences provide insight into *Plasmodium vivax* and the monkey malaria clade. Nat Genet. 2012; 44:1051–5.

Volkman SK, Sabeti PC, DeCaprio D, Neafsey DE, Schaffner SF, Milner Jr DA, et al. A genome-wide map of diversity in *Plasmodium falciparum*. Nat Genet. 2007; 39:113–9.

**Table S1**. Samples used in this study for multi-locus species delimitation. Shown below are sample code, AMCC tissue number from the American Museum of Natural History, *cytb* haplotype name (from MalAvi database, <http://mbio-serv2.mbioekol.lu.se/Malavi/>), host species from which the *Leucocytozoon* haplotype was isolated, host family of that sample, and sampling site (see Fig. 1 from main paper body).

| **Sample code** | **AMNH DOT**  **Specimen Number** | **Cytb haplotype**  **(MalAvi name)** | **Host species** | **Host Family** | **Sampling Site** |
| --- | --- | --- | --- | --- | --- |
| PRS4416 | 23236 | ACAFLA03 | *Loxia leucoptera* | Fringillidae | Denali 1 (63.32385, -148.23181) |
| PRS4456 | 23276 | ACAFLA03 | *Acanthis flammea* | Fringillidae | Denali 3 (63.07621, -146.22423) |
| JEL269 | 23777 | ACAFLA03 | *Acanthis flammea* | Fringillidae | Denali 1 (63.32385, -148.23181) |
| JEL276 | 23784 | ACAFLA03 | *Acanthis flammea* | Fringillidae | Denali 2 (63.03881, -147.00372) |
| PRS4472 | 23808 | ACAFLA03 | *Acanthis flammea* | Fringillidae | Elliot 1 (65.28899, -149.29083) |
| PRS4482 | 23818 | ACAFLA03 | *Setophaga coronata* | Parulidae | Elliot 1 (65.28899, -149.29083) |
| PRS4483 | 23819 | ACAFLA03 | *Junco hyemalis* | Emberizidae | Elliot 1 (65.28899, -149.29083) |
| PRS4517 | 23853 | ACAFLA04 | *Acanthis flammea* | Fringillidae | Denali 1 (63.32385, -148.23181) |
| SCG268 | 23334 | ACAFLA01 | *Acanthis flammea* | Fringillidae | Denali 2 (63.03881, -147.00372) |
| PRS4514 | 23850 | BOMGAR01 | *Bombycilla garrulus* | Bombycillidae | Denali 1 (63.32385, -148.23181) |
| SCG277 | 23343 | BT1 | *Phylloscopus borealis* | Phylloscopidae | Denali 2 (63.03881, -147.00372) |
| PRS4439 | 23259 | BT1 | *Phylloscopus borealis* | Phylloscopidae | Denali 2 (63.03881, -147.00372) |
| SCG280 | 23346 | BT1 | *Phylloscopus borealis* | Phylloscopidae | Denali 3 (63.07621, -146.22423) |
| GFB3848 | 23719 | CARPUS01 | *Cardellina pusilla* | Parulidae | Denali 2 (63.03881, -147.00372) |
| PRS4450 | 23270 | CATGUT02 | *Catharus minimus* | Turdidae | Denali 3 (63.07621, -146.22423) |
| PRS4441 | 23261 | CATGUT02 | *Catharus minimus* | Turdidae | Denali 2 (63.03881, -147.00372) |
| GFB3815 | 23688 | CATGUT02 | *Catharus minimus* | Turdidae | Denali 1 (63.32385, -148.23181) |
| GFB3855 | 23726 | CATGUT02 | *Catharus minimus* | Turdidae | Denali 2 (63.03881, -147.00372) |
| PRS4543 | 23879 | CATGUT02 | *Catharus minimus* | Turdidae | Denali 2 (63.03881, -147.00372) |
| PRS4452 | 23272 | CATMIN01 | *Catharus minimus* | Turdidae | Denali 3 (63.07621, -146.22423) |
| JEL252 | 23760 | CATMIN05 | *Catharus ustulatus* | Turdidae | Elliot 1 (65.28899, -149.29083) |
| PRS4512 | 23848 | CATUST09 | *Catharus minimus* | Turdidae | Denali 1 (63.32385, -148.23181) |
| PRS4389 | 23209 | CATUST09 | *Catharus ustulatus* | Turdidae | Steese 2 (65.163393, -147.321237) |
| JEL251 | 23759 | CATUST09 | *Catharus minimus* | Turdidae | Elliot 1 (65.28899, -149.29083) |
| PRS4457 | 23277 | CATUST14 | *Anthus rubescens* | Motacillidae | Denali 3 (63.07621, -146.22423) |
| PRS4508 | 23844 | CATUST14 | *Regulus calendula* | Regulidae | Denali 1 (63.32385, -148.23181) |
| GFB3811 | 23684 | CATUST28 | *Catharus ustulatus* | Turdidae | Denali 3 (63.07621, -146.22423) |
| PRS4418 | 23238 | CB1 | *Loxia leucoptera* | Fringillidae | Denali 1 (63.32385, -148.23181) |
| SCG223 | 23290 | CB1 | *Cardellina pusilla* | Parulidae | Steese 1 (65.23043, -146.94366) |
| SCG234 | 23301 | CB1 | *Poecile hudsonicus* | Paridae | Steese 2 (65.163393, -147.321237) |
| SCG282 | 23348 | CB1 | *Zonotrichia leucophrys* | Emberizidae | Denali 3 (63.07621, -146.22423) |
| JEL232 | 23740 | CB1 | *Junco hyemalis* | Emberizidae | Elliot 1 (65.28899, -149.29083) |
| JEL235 | 23743 | CB1 | *Oreothlypis celata* | Parulidae | Elliot 1 (65.28899, -149.29083) |
| JEL244 | 23752 | CB1 | *Oreothlypis celata* | Parulidae | Elliot 1 (65.28899, -149.29083) |
| JEL245 | 23753 | CB1 | *Oreothlypis celata* | Parulidae | Elliot 1 (65.28899, -149.29083) |
| PRS4476 | 23812 | CB1 | *Zonotrichia leucophrys* | Emberizidae | Elliot 1 (65.28899, -149.29083) |
| PRS4496 | 23832 | CB1 | *Oreothlypis celata* | Parulidae | Elliot 1 (65.28899, -149.29083) |
| SCG231 | 23298 | CNEORN01 | *Setophaga townsendi* | Parulidae | Steese 2 (65.163393, -147.321237) |
| SCG269 | 23335 | CNEORN01 | *Cardellina pusilla* | Parulidae | Denali 2 (63.03881, -147.00372) |
| GFB3779 | 23653 | CNEORN01 | *Setophaga coronata* | Parulidae | Elliot 1 (65.28899, -149.29083) |
| GFB3788 | 23662 | CNEORN01 | *Setophaga coronata* | Parulidae | Elliot 1 (65.28899, -149.29083) |
| JEL289 | 23797 | CNEORN01 | *Cardellina pusilla* | Parulidae | Denali 2 (63.03881, -147.00372) |
| PRS4432 | 23252 | COBRA07 | *Pica hudsonia* | Corvidae | Denali 1 (63.32385, -148.23181) |
| PRS4387 | 23207 | JUNHYE04 | *Junco hyemalis* | Emberizidae | Steese 2 (65.163393, -147.321237) |
| PRS4414 | 23234 | LOXLEU05 | *Loxia leucoptera* | Fringillidae | Denali 1 (63.32385, -148.23181) |
| PRS4372 | 23192 | PERCAN01 | *Perisoreus canadensis* | Corvidae | Steese 1 (65.23043, -146.94366) |
| PRS4392 | 23212 | PERCAN01 | *Perisoreus canadensis* | Corvidae | Steese 2 (65.163393, -147.321237) |
| SCG233 | 23300 | PERCAN01 | *Setophaga coronata* | Parulidae | Steese 2 (65.163393, -147.321237) |
| PRS4497 | 23833 | PERCAN01 | *Perisoreus canadensis* | Corvidae | Elliot 1 (65.28899, -149.29083) |
| GFB3797 | 23671 | PERCAN01 | *Junco hyemalis* | Emberizidae | Elliot 1 (65.28899, -149.29083) |
| JEL249 | 23757 | PERCAN01 | *Perisoreus canadensis* | Corvidae | Elliot 1 (65.28899, -149.29083) |
| PRS4431 | 23251 | PERCAN01 | *Perisoreus canadensis* | Corvidae | Denali 1 (63.32385, -148.23181) |
| JEL230 | 23738 | PERCAN04 | *Setophaga coronata* | Parulidae | Elliot 1 (65.28899, -149.29083) |
| SCG267 | 23333 | PHYBOR01 | *Phylloscopus borealis* | Phylloscopidae | Denali 2 (63.03881, -147.00372) |
| PRS4458 | 23278 | ROFI06 | *Acanthis flammea* | Fringillidae | Denali 3 (63.07621, -146.22423) |
| PRS4448 | 23268 | ROFI06 | *Acanthis flammea* | Fringillidae | Denali 2 (63.03881, -147.00372) |
| GFB3781 | 23655 | ROFI06 | *Acanthis flammea* | Fringillidae | Elliot 1 (65.28899, -149.29083) |
| SCG271 | 23337 | SETPET04 | *Setophaga petechia* | Parulidae | Denali 2 (63.03881, -147.00372) |
| SCG246 | 23313 | TRPIP2 | *Loxia leucoptera* | Fringillidae | Denali 1 (63.32385, -148.23181) |
| PRS4424 | 23244 | TUMIG11 | *Turdus migratorius* | Turdidae | Denali 1 (63.32385, -148.23181) |
| PRS4404 | 23224 | TUMIG15 | *Turdus migratorius* | Turdidae | Steese 2 (65.163393, -147.321237) |
| PRS4502 | 23838 | TUMIG20 | *Turdus migratorius* | Turdidae | Denali 1 (63.32385, -148.23181) |
| PRS4379 | 23199 | ZOLEU02 | *Junco hyemalis* | Emberizidae | Steese 1 (65.23043, -146.94366) |
| PRS4438 | 23258 | ZOLEU02 | *Spizella arborea* | Emberizidae | Denali 2 (63.03881, -147.00372) |
| SCG220 | 23287 | ZOLEU02 | *Melospiza lincolnii* | Emberizidae | Steese 1 (65.23043, -146.94366) |
| GFB3777 | 23651 | ZOLEU02 | *Junco hyemalis* | Emberizidae | Elliot 1 (65.28899, -149.29083) |
| JEL242 | 23750 | ZOLEU02 | *Junco hyemalis* | Emberizidae | Elliot 1 (65.28899, -149.29083) |
| PRS4468 | 23804 | ZOLEU02 | *Junco hyemalis* | Emberizidae | Elliot 1 (65.28899, -149.29083) |
| SCG230 | 23297 | ZOLEU04 | *Zonotrichia leucophrys* | Emberizidae | Steese 1 (65.23043, -146.94366) |

**Table S2**. Genes amplified in this study. All loci were amplified using the PCR protocol used in Borner et al. (2016). All inner primers included CAG and M13 tags (denoted in **red** in primer sequence below) to improve sequencing efficiency.

| **Gene** | **Primers** | **Notes** |
| --- | --- | --- |
| *ATPase2* | Outer Forward:  **GCWGGWATTCATATATGGATGYT** |  |
|  | Outer Reverse:  **TCATTHGCWCCATCTCCWATWGC** |  |
|  | Inner Forward:  **CAGTCGGGCGTCATCAGCWGGWATTCATATATGGATGYT** |  |
|  | Inner Reverse:  **GGAAACAGCTATGACCATCACAHGTACATTTATCWGCTAA** |  |
| *RPB1* | Outer Forward:  **GWATWCAATTAACAAATATWCAAGT** |  |
|  | Outer Reverse:  **GGYTCTTCTCTCATATATACTTTTGT** |  |
|  | Inner Forward:  **CAGTCGGGCGTCATCAATATTCYGTATTTTCRAGAGATG** |  |
|  | Inner Reverse:  **GGAAACAGCTATGACCATTACTTTTGTWATATTHTCWATACCT** |  |
| *POLD1* | Outer Forward:  **ACTCTYGATTTTGCWTCYTTRT** | Has potential to preferentially amplify *Parahaemoproteus* infections when parasitemia of *Parahaemoproteus* is significantly higher than *Leucocytozoon* |
|  | Outer Reverse:  **TTCRATHCCTTTACARTCCATT** |  |
|  | Inner Forward:  **CAGTCGGGCGTCATCATCCATCWATTATGATWGCAC** |  |
|  | Inner Reverse:  **GGAAACAGCTATGACCATTTKDGCWGCTTCTTTTCCTA** |  |
| *KPNB1* | Outer Forward:  **TGGAAGGWCCWGAYACWGATAA** |  |
|  | Outer Reverse:  **TGCATDGTHCCACAATAATAYCC** |  |
|  | Inner Forward:  **CAGTCGGGCGTCATCACWGAYACWGATAAATTAAARC** |  |
|  | Inner Reverse:  **GGAAACAGCTATGACCATATGKGATAATAAYTCWATCA** |  |
| *PRPF6* | Outer Forward:  **TGGATATACGAAGAAAAAGTAGA** | Apparent primer bias against *Leucocytozoon* in clade 2 (Fig. 2) |
|  | Outer Reverse:  **CCTCTKGCTTCTCCYACATCA** |  |
|  | Inner Forward:  **CAGTCGGGCGTCATCACCAACWATTCAAGARCAATTTAGTG** |  |
|  | Inner Reverse:  **GGAAACAGCTATGACCATGGAGTTTGTAATCCTAAGCCTAA** |  |
| *SEC24a* | Outer Forward:  **TGCRGTKAATAGTGGWTTRT** |  |
|  | Outer Reverse:  **GATTCCCAWGCWGTTTCWGT** |  |
|  | Inner Forward:  **CAGTCGGGCGTCATCAKAATAGTGGWTTRTTRGATA** |  |
|  | Inner Reverse:  **GGAAACAGCTATGACCATTGGACAWGCAAAYAAATCTA** |  |
| *SF3B1* | Outer Forward:  **TAGTACWWCWGATGAAGAAATGAA** | Apparent primer bias against *Leucocytozoon* in clade 1 (Fig. 2) |
|  | Outer Reverse:  **WATTCTTGATATTAAATCTGCAC** |  |
|  | Inner Forward:  **CAGTCGGGCGTCATCAAACAATGTATACARACWGAAGG** |  |
|  | Inner Reverse:  **GGAAACAGCTATGACCATCTTATWATWCCWGCTATYTGWGGTA** |  |

**Table S3**. **Summary of *Leucocytozoon* *cytb* haplotypes included in species delimitation analyses.** Shown are the number of times each haplotype was detected across all 381 hosts that were surveyed and the number of single-infection samples that were used for multi-locus species delimitation analyses. Note that the total number of detections (406) is greater than the number of hosts surveyed (381) due to the presence of mixed infections in some surveyed hosts.

| ***Cytb* haplotype** | **N (total detections)** | **N (species delimitation)** |
| --- | --- | --- |
| ACAFLA01 | 1 | 1 |
| ACAFLA03 | 29 | 7 |
| ACAFLA04 | 1 | 1 |
| BOMGAR01 | 1 | 1 |
| BT1 | 4 | 3 |
| CARPUS01 | 1 | 1 |
| CATGUT02 | 11 | 5 |
| CATMIN01 | 10 | 1 |
| CATMIN05 | 15 | 1 |
| CATUST09 | 27 | 3 |
| CATUST14 | 11 | 2 |
| CATUST28 | 7 | 1 |
| CB1 | 100 | 10 |
| CNEORN01 | 43 | 5 |
| COBRA07 | 1 | 1 |
| JUNHYE04 | 1 | 1 |
| LOXLEU05 | 4 | 1 |
| PERCAN01 | 11 | 7 |
| PERCAN04 | 7 | 1 |
| PHYBOR01 | 1 | 1 |
| ROFI06 | 22 | 3 |
| SETPET04 | 1 | 1 |
| TRPIP2 | 12 | 1 |
| TUMIG11 | 5 | 1 |
| TUMIG15 | 2 | 1 |
| TUMIG20 | 1 | 1 |
| ZOLEU02 | 76 | 6 |
| ZOLEU04 | 1 | 1 |

**Table S4.** GenBank accession numbers for genetic data collected in this study. GenBank numbers for ATPase2: MG946320 - MG946383, RPB1: MG946384 - MG946450, POLD1: MG946451 - MG946505, KPNB1: MG946506 - MG946572, PRPF6: MG946573 - MG946614, SEC24a: MG946615 - MG946674, SF3B1: MG946675 - MG946714. Note that samples 243938 and 243938 were removed from species delimitation analyses.

| **AMNH DOT**  **Specimen Number** | **Cytb lineage**  **(MalAvi name)** | ***Cytb* accession** | ***ATPase2***  **accession** | ***RPB1* accession** | ***POLD1* accession** | ***KPNB1* accession** | ***PRPF6* accession** | ***SEC24a* accession** | ***SF3B1* accession** |
| --- | --- | --- | --- | --- | --- | --- | --- | --- | --- |
| 23236 | ACAFLA03 | MG726132 |  | MG946411 | MG946467 | MG946546 | MG946599 | MG946647 |  |
| 23276 | ACAFLA03 | - | MG946361 | MG946422 | MG946484 | MG946541 | MG946600 | MG946652 |  |
| 23777 | ACAFLA03 | - | MG946350 | MG946401 | MG946465 | MG946530 | MG946597 | MG946637 |  |
| 23784 | ACAFLA03 | - | MG946349 | MG946402 | MG946466 |  | MG946598 | MG946638 |  |
| 23808 | ACAFLA03 | - | MG946353 | MG946426 | MG946485 | MG946545 | MG946601 |  |  |
| 23818 | ACAFLA03 | - | MG946351 | MG946428 | MG946486 | MG946547 | MG946602 | MG946639 |  |
| 23819 | ACAFLA03 | - | MG946352 | MG946429 | MG946487 | MG946548 | MG946603 | MG946640 |  |
| 23853 | ACAFLA04 | MG726106 | MG946381 |  |  | MG946558 |  |  |  |
| 23334 | ACAFLA01 | MG726100 | MG946372 | MG946444 | MG946497 | MG946566 |  | MG946667 | MG946708 |
| 23850 | BOMGAR01 | MG726134 | MG946369 | MG946435 | MG946490 | MG946557 | MG946606 | MG946661 |  |
| 23343 | BT1 | MG726108 | MG946383 | MG946447 | MG946502 | MG946569 |  | MG946672 | MG946711 |
| 23259 | BT1 | - | MG946359 | MG946417 | MG946479 | MG946568 |  | MG946650 | MG946698 |
| 23346 | BT1 | - | MG946374 | MG946448 | MG946503 | MG946570 |  | MG946673 | MG946712 |
| 23719 | CARPUS01 | MG726107 | MG946376 | MG946391 | MG946457 | MG946515 |  | MG946625 | MG946679 |
| 23270 | CATGUT02 | MG726116 | MG946365 | MG946420 |  | MG946518 |  |  | MG946700 |
| 23261 | CATGUT02 | - | MG946380 | MG946418 |  | MG946520 | MG946607 | MG946618 | MG946699 |
| 23688 | CATGUT02 | - | MG946364 | MG946390 |  | MG946516 |  | MG946617 | MG946678 |
| 23726 | CATGUT02 | - | MG946366 | MG946392 |  | MG946517 |  | MG946620 | MG946680 |
| 23879 | CATGUT02 | - | MG946367 | MG946436 |  | MG946519 | MG946608 | MG946619 | MG946704 |
| 23272 | CATMIN01 | MG726151 | MG946360 | MG946421 | MG946480 | MG946540 | MG946593 |  | MG946701 |
| 23760 | CATMIN05 | MG726119 | MG946348 | MG946400 | MG946464 | MG946529 |  | MG946636 | MG946689 |
| 23848 | CATUST09 | MG726163 |  | MG946434 |  | MG946556 |  | MG946660 |  |
| 23209 | CATUST09 | - | MG946356 | MG946407 | MG946471 | MG946533 | MG946582 | MG946658 | MG946692 |
| 23759 | CATUST09 | - | MG946355 | MG946399 | MG946470 | MG946528 | MG946581 | MG946659 | MG946688 |
| 23277 | CATUST14 | MG726129 | MG946362 | MG946423 | MG946481 | MG946542 | MG946594 | MG946653 |  |
| 23844 | CATUST14 | - | MG946363 | MG946433 |  | MG946543 | MG946595 |  |  |
| 23684 | CATUST28 | MG726118 |  | MG946389 | MG946456 | MG946514 |  | MG946624 |  |
| 23238 | CB1 | MG726102 | MG946342 | MG946412 | MG946475 | MG946551 |  | MG946629 | MG946694 |
| 23290 | CB1 | - | MG946347 | MG946437 | MG946492 | MG946560 |  | MG946631 | MG946686 |
| 23301 | CB1 | - | MG946345 | MG946441 | MG946494 | MG946563 |  | MG946664 |  |
| 23348 | CB1 | - | MG946346 | MG946449 | MG946504 | MG946572 |  | MG946668 | MG946713 |
| 23740 | CB1 | - | MG946338 |  | MG946460 | MG946549 |  | MG946626 | MG946681 |
| 23743 | CB1 | - | MG946339 | MG946394 |  | MG946550 |  | MG946627 | MG946682 |
| 23752 | CB1 | - | MG946340 | MG946396 | MG946461 | MG946523 |  | MG946628 | MG946683 |
| 23753 | CB1 | - | MG946341 | MG946397 | MG946462 | MG946524 |  |  | MG946684 |
| 23812 | CB1 | - | MG946343 | MG946427 | MG946483 | MG946552 |  | MG946630 | MG946685 |
| 23832 | CB1 | - | MG946344 | MG946430 | MG946488 | MG946553 |  | MG946632 | MG946687 |
| 23298 | CNEORN01 | MG726148 | MG946382 | MG946439 |  | MG946509 | MG946609 | MG946663 | MG946705 |
| 23335 | CNEORN01 | - | MG946373 | MG946445 | MG946499 | MG946510 | MG946584 | MG946669 | MG946709 |
| 23653 | CNEORN01 | - | MG946328 | MG946385 |  | MG946506 | MG946577 | MG946615 | MG946675 |
| 23662 | CNEORN01 | - | MG946327 | MG946387 |  | MG946507 | MG946578 | MG946616 | MG946677 |
| 23797 | CNEORN01 | - | MG946329 | MG946403 | MG946498 | MG946508 | MG946583 | MG946641 | MG946690 |
| 23252 | COBRA07 | MG726111 | MG946358 | MG946415 |  | MG946538 |  |  | MG946696 |
| 23207 | JUNHYE04 | MG726140 | MG946377 | MG946406 |  | MG946532 |  | MG946643 | MG946691 |
| 23234 | LOXLEU05 | MG726137 | MG946379 | MG946410 | MG946474 | MG946537 | MG946590 | MG946646 | MG946693 |
| 23192 | PERCAN01 | MG726130 | MG946335 | MG946404 | MG946468 | MG946526 | MG946586 | MG946635 |  |
| 23212 | PERCAN01 | - | MG946378 | MG946408 | MG946472 | MG946527 | MG946588 | MG946644 |  |
| 23300 | PERCAN01 | - | MG946336 | MG946440 | MG946493 | MG946562 | MG946612 | MG946656 |  |
| 23833 | PERCAN01 | - | MG946368 | MG946431 | MG946489 | MG946554 | MG946604 | MG946655 |  |
| 23671 | PERCAN01 | - | MG946333 | MG946388 | MG946455 | MG946513 |  | MG946623 |  |
| 23757 | PERCAN01 | - | MG946334 | MG946398 | MG946463 | MG946525 | MG946580 | MG946634 |  |
| 23251 | PERCAN01 | - | MG946354 | MG946414 | MG946477 |  | MG946591 | MG946648 |  |
| 23738 | PERCAN04 | MG726112 | MG946337 | MG946393 | MG946458 | MG946521 |  |  |  |
| 23333 | PHYBOR01 | MG726120 | MG946371 | MG946442 | MG946496 | MG946565 |  | MG946665 | MG946707 |
| 23278 | ROFI06 | MG726136 | MG946332 | MG946424 | MG946454 | MG946536 | MG946575 | MG946654 | MG946703 |
| 23268 | ROFI06 | - | MG946330 | MG946419 | MG946453 | MG946535 | MG946574 | MG946651 | MG946702 |
| 23655 | ROFI06 | - | MG946331 | MG946386 | MG946452 | MG946512 | MG946573 | MG946622 | MG946676 |
| 23337 | SETPET04 | MG726150 |  |  | MG946501 | MG946567 | MG946585 | MG946670 | MG946710 |
| 23313 | TRPIP2 | MG726099 | MG946370 | MG946443 | MG946495 | MG946564 |  | MG946666 | MG946706 |
| 23244 | TUMIG11 | MG726115 | MG946357 | MG946413 | MG946476 |  |  |  | MG946695 |
| 23224 | TUMIG15 | MG726122 |  | MG946409 | MG946473 | MG946534 | MG946589 | MG946645 |  |
| 23838 | TUMIG20 | MG726114 |  | MG946432 |  | MG946555 | MG946605 | MG946657 |  |
| 23199 | ZOLEU02 | MG726144 | MG946322 | MG946405 | MG946469 | MG946531 | MG946587 | MG946642 |  |
| 23258 | ZOLEU02 | - | MG946325 | MG946416 | MG946478 | MG946539 | MG946592 | MG946649 | MG946697 |
| 23287 | ZOLEU02 | - | MG946323 |  | MG946491 | MG946559 | MG946610 | MG946662 |  |
| 23651 | ZOLEU02 | - | MG946326 | MG946384 | MG946451 | MG946511 | MG946576 | MG946621 |  |
| 23750 | ZOLEU02 | - | MG946324 | MG946395 | MG946459 | MG946522 | MG946579 | MG946633 |  |
| 23804 | ZOLEU02 | - | MG946320 | MG946425 | MG946482 | MG946544 | MG946596 |  |  |
| 23297 | ZOLEU04 | MG726147 | MG946321 |  |  | MG946561 | MG946611 |  |  |
| 23258 | SPIARB01 | MG726177 | MG946375 | MG946450 | MG946505 | MG946571 | MG946614 | MG946674 | MG946714 |

**Table S5.** Pairwise uncorrected genetic divergence (%) among samples of the haplotype PERCAN01 for six loci sequenced in this study (samples of this haplotype did not amplify for locus SF3B1). One sample, PRS4431, exhibited unusually high genetic divergence from all other samples of this haplotype for the loci ATPase2 and PRPF6 and so it was removed from further analysis.

| **ATPase2** |  |  |  |  |  |  |  |
| --- | --- | --- | --- | --- | --- | --- | --- |
|  | GFB3797  PERCAN01 | JEL249  PERCAN01 | PRS4372  PERCAN01 | SCG233  PERCAN01 | PRS4431  PERCAN01 | PRS4497  PERCAN01 | PRS4392  PERCAN01 |
| GFB3797  PERCAN01 | 0 |  |  |  |  |  |  |
| JEL249  PERCAN01 | 0 | 0 |  |  |  |  |  |
| PRS4372  PERCAN01 | 0 | 0 | 0 |  |  |  |  |
| SCG233  PERCAN01 | 0.35 | 0.35 | 0.35 | 0 |  |  |  |
| PRS4431  PERCAN01 | 8.2 | 8.2 | 8.2 | 8.2 | 0 |  |  |
| PRS4497  PERCAN01 | 0 | 0 | 0 | 0.35 | 8.2 | 0 |  |
| PRS4392  PERCAN01 | 0.35 | 0.35 | 0.35 | 0 | 8.2 | 0.35 | 0 |
|  |  |  |  |  |  |  |  |
| **RPB1** |  |  |  |  |  |  |  |
|  | GFB3797  PERCAN01 | JEL249  PERCAN01 | PRS4372  PERCAN01 | PRS4392  PERCAN01 | PRS4431  PERCAN01 | PRS4497  PERCAN01 | SCG233  PERCAN01 |
| GFB3797  PERCAN01 | 0 |  |  |  |  |  |  |
| JEL249  PERCAN01 | 0 | 0 |  |  |  |  |  |
| PRS4372  PERCAN01 | 0 | 0 | 0 |  |  |  |  |
| PRS4392  PERCAN01 | 0 | 0 | 0 | 0 |  |  |  |
| PRS4431  PERCAN01 | 0 | 0 | 0 | 0 | 0 |  |  |
| PRS4497  PERCAN01 | 0 | 0 | 0 | 0 | 0 | 0 |  |
| SCG233  PERCAN01 | 0 | 0 | 0 | 0 | 0 | 0 | 0 |
|  |  |  |  |  |  |  |  |
| **POLD1** |  |  |  |  |  |  |  |
|  | GFB3797  PERCAN01 | JEL249  PERCAN01 | PRS4372  PERCAN01 | PRS4392  PERCAN01 | PRS4431  PERCAN01 | PRS4497  PERCAN01 | SCG233  PERCAN01 |
| GFB3797  PERCAN01 | 0 |  |  |  |  |  |  |
| JEL249  PERCAN01 | 0 | 0 |  |  |  |  |  |
| PRS4372  PERCAN01 | 0 | 0 | 0 |  |  |  |  |
| PRS4392  PERCAN01 | 0 | 0 | 0 | 0 |  |  |  |
| PRS4431  PERCAN01 | 0 | 0 | 0 | 0 | 0 |  |  |
| PRS4497  PERCAN01 | 0 | 0 | 0 | 0 | 0 | 0 |  |
| SCG233  PERCAN01 | 0 | 0 | 0 | 0 | 0 | 0 | 0 |
|  |  |  |  |  |  |  |  |
| **KPBN1** |  |  |  |  |  |  |  |
|  | GFB3797  PERCAN01 | JEL249  PERCAN01 | PRS4372  PERCAN01 | PRS4392  PERCAN01 | PRS4497  PERCAN01 | SCG233  PERCAN01 |  |
| GFB3797  PERCAN01 | 0 |  |  |  |  |  |  |
| JEL249  PERCAN01 | 0 | 0 |  |  |  |  |  |
| PRS4372  PERCAN01 | 0 | 0 | 0 |  |  |  |  |
| PRS4392  PERCAN01 | 0 | 0 | 0 | 0 |  |  |  |
| PRS4497  PERCAN01 | 0 | 0 | 0 | 0 | 0 |  |  |
| SCG233  PERCAN01 | 0 | 0 | 0 | 0 | 0 | 0 |  |
|  |  |  |  |  |  |  |  |
| **PRPF6** |  |  |  |  |  |  |  |
|  | JEL249  PERCAN01 | PRS4372  PERCAN01 | PRS4392  PERCAN01 | PRS4431  PERCAN01 | PRS4497  PERCAN01 | SCG233  PERCAN01 |  |
| JEL249  PERCAN01 | 0 |  |  |  |  |  |  |
| PRS4372  PERCAN01 | 0 | 0 |  |  |  |  |  |
| PRS4392  PERCAN01 | 0 | 0 | 0 |  |  |  |  |
| PRS4431  PERCAN01 | 9.3 | 9.3 | 9.3 | 0 |  |  |  |
| PRS4497  PERCAN01 | 0 | 0 | 0 | 9.3 | 0 |  |  |
| SCG233  PERCAN01 | 0 | 0 | 0 | 9.3 | 0 | 0 |  |
|  |  |  |  |  |  |  |  |
| **SEC24A** |  |  |  |  |  |  |  |
|  | GFB3797  PERCAN01 | JEL249  PERCAN01 | PRS4372  PERCAN01 | PRS4392  PERCAN01 | PRS4431  PERCAN01 | PRS4497  PERCAN01 | SCG233  PERCAN01 |
| GFB3797  PERCAN01 | 0 |  |  |  |  |  |  |
| JEL249  PERCAN01 | 0 | 0 |  |  |  |  |  |
| PRS4372  PERCAN01 | 0 | 0 | 0 |  |  |  |  |
| PRS4392  PERCAN01 | 0 | 0 | 0 | 0 |  |  |  |
| PRS4431  PERCAN01 | 0 | 0 | 0 | 0 | 0 |  |  |
| PRS4497  PERCAN01 | 0 | 0 | 0 | 0 | 0 | 0 |  |
| SCG233  PERCAN01 | 0 | 0 | 0 | 0 | 0 | 0 | 0 |

**Table S6.** Pairwise uncorrected genetic divergence (%) among samples of the haplotype ACAFLA03 for six loci sequenced in this study (samples of this haplotype did not amplify for locus SF3B1). One sample, PRS4416, exhibited unusually high genetic divergence from all other samples of this haplotype for the locus POLD1 and so it was removed from further analysis.

| **ATPase2** |  |  |  |  |  |  |  |
| --- | --- | --- | --- | --- | --- | --- | --- |
|  | JEL276  ACAFLA03 | JEL269  ACAFLA03 | PRS4482  ACAFLA03 | PRS4483  ACAFLA03 | PRS4472  ACAFLA03 | PRS4456  ACAFLA03 | |
| JEL276  ACAFLA03 | 0 |  |  |  |  |  |  |
| JEL269  ACAFLA03 | 0.62 | 0 |  |  |  |  |  |
| PRS4482  ACAFLA03 | 0.62 | 0 | 0 |  |  |  |  |
| PRS4483  ACAFLA03 | 0.62 | 0 | 0 | 0 |  |  |  |
| PRS4472  ACAFLA03 | 0.62 | 0 | 0 | 0 | 0 |  |  |
| PRS4456  ACAFLA03 | 0.62 | 0 | 0 | 0 | 0 | 0 |  |
|  |  |  |  |  |  |  |  |
| **RPB1** |  |  |  |  |  |  |  |
|  | JEL269  ACAFLA03 | JEL276  ACAFLA03 | PRS4416  ACAFLA03 | PRS4456  ACAFLA03 | PRS4472  ACAFLA03 | PRS4482  ACAFLA03 | PRS4483  ACAFLA03 |
| JEL269  ACAFLA03 | 0 |  |  |  |  |  |  |
| JEL276  ACAFLA03 | 0 | 0 |  |  |  |  |  |
| PRS4416  ACAFLA03 | 0 | 0 | 0 |  |  |  |  |
| PRS4456  ACAFLA03 | 0 | 0 | 0 | 0 |  |  |  |
| PRS4472  ACAFLA03 | 0 | 0 | 0 | 0 | 0 |  |  |
| PRS4482  ACAFLA03 | 0 | 0 | 0 | 0 | 0 | 0 |  |
| PRS4483  ACAFLA03 | 0 | 0 | 0 | 0 | 0 | 0 | 0 |
|  |  |  |  |  |  |  |  |
| **POLD1** |  |  |  |  |  |  |  |
|  | JEL269  ACAFLA03 | JEL276  ACAFLA03 | PRS4416  ACAFLA03 | PRS4456  ACAFLA03 | PRS4472  ACAFLA03 | PRS4482  ACAFLA03 | PRS4483  ACAFLA03 |
| JEL269  ACAFLA03 | 0 |  |  |  |  |  |  |
| JEL276  ACAFLA03 | 0 | 0 |  |  |  |  |  |
| PRS4416  ACAFLA03 | 5.8 | 5.8 | 0 |  |  |  |  |
| PRS4456  ACAFLA03 | 0 | 0 | 5.8 | 0 |  |  |  |
| PRS4472  ACAFLA03 | 0 | 0 | 5.8 | 0 | 0 |  |  |
| PRS4482  ACAFLA03 | 0 | 0 | 5.8 | 0 | 0 | 0 |  |
| PRS4483  ACAFLA03 | 0 | 0 | 5.8 | 0 | 0 | 0 | 0 |
|  |  |  |  |  |  |  |  |
| **KPBN1** |  |  |  |  |  |  |  |
|  | JEL269  ACAFLA03 | PRS4456  ACAFLA03 | PRS4472  ACAFLA03 | PRS4416  ACAFLA03 | PRS4482  ACAFLA03 | PRS4483  ACAFLA03 | |
| JEL269  ACAFLA03 | 0 |  |  |  |  |  |  |
| PRS4456  ACAFLA03 | 0 | 0 |  |  |  |  |  |
| PRS4472  ACAFLA03 | 0 | 0 | 0 |  |  |  |  |
| PRS4416  ACAFLA03 | 0 | 0 | 0 | 0 |  |  |  |
| PRS4482  ACAFLA03 | 0 | 0 | 0 | 0 | 0 |  |  |
| PRS4483  ACAFLA03 | 0 | 0 | 0 | 0 | 0 | 0 |  |
|  |  |  |  |  |  |  |  |
| **PRPF6** |  |  |  |  |  |  |  |
|  | JEL269  ACAFLA03 | JEL276  ACAFLA03 | PRS4416  ACAFLA03 | PRS4456  ACAFLA03 | PRS4472  ACAFLA03 | PRS4482  ACAFLA03 | PRS4483  ACAFLA03 |
| JEL269  ACAFLA03 | 0 |  |  |  |  |  |  |
| JEL276  ACAFLA03 | 0.44 | 0 |  |  |  |  |  |
| PRS4416  ACAFLA03 | 0 | 0.44 | 0 |  |  |  |  |
| PRS4456  ACAFLA03 | 0 | 0.44 | 0 | 0 |  |  |  |
| PRS4472  ACAFLA03 | 0.44 | 0.88 | 0.44 | 0.44 | 0 |  |  |
| PRS4482  ACAFLA03 | 0 | 0.44 | 0 | 0 | 0.44 | 0 |  |
| PRS4483  ACAFLA03 | 0.44 | 0.88 | 0.44 | 0.44 | 0 | 0.44 | 0 |
|  |  |  |  |  |  |  |  |
| **SEC24A** |  |  |  |  |  |  |  |
|  | JEL269  ACAFLA03 | JEL276  ACAFLA03 | PRS4482  ACAFLA03 | PRS4483  ACAFLA03 | PRS4416  ACAFLA03 | PRS4456  ACAFLA03 | |
| JEL269  ACAFLA03 | 0 |  |  |  |  |  |  |
| JEL276  ACAFLA03 | 0 | 0 |  |  |  |  |  |
| PRS4482  ACAFLA03 | 0 | 0 | 0 |  |  |  |  |
| PRS4483  ACAFLA03 | 0 | 0 | 0 | 0 |  |  |  |
| PRS4416  ACAFLA03 | 0 | 0 | 0 | 0 | 0 |  |  |
| PRS4456  ACAFLA03 | 0 | 0 | 0 | 0 | 0 | 0 |  |

**Table S7.** Support values for bGMYC analysis.

| **Analysis** | **Probability** | **Species** |
| --- | --- | --- |
| bGMYC | 0.9983762 | GFB3815_CATGUT02, GFB3855_CATGUT02, PRS4441_CATGUT02, PRS4450_CATGUT02, PRS4543_CATGUT02 |
| bGMYC | 0.9982574 | PRS4404_TUMIG15 |
| bGMYC | 0.9980594 | PRS4452_CATMIN01 |
| bGMYC | 0.9954455 | PRS4424_TUMIG11 |
| bGMYC | 0.9954455 | PRS4502_TUMIG20 |
| bGMYC | 0.9945743 | JEL251_CATUST09, PRS4389_CATUST09, PRS4512_CATUST09 |
| bGMYC | 0.9929505 | JEL230_PERCAN04 |
| bGMYC | 0.9929505 | PRS4432_COBRA07 |
| bGMYC | 0.9924356 | GFB3777_ZOLEU02, JEL242_ZOLEU02, PRS4379_ZOLEU02, PRS4438_ZOLEU02, PRS4468_ZOLEU02, SCG220_ZOLEU02, SCG230_ZOLEU04 |
| bGMYC | 0.9914059 | SCG246_TRPIP2, SCG268_ACFLA01 |
| bGMYC | 0.990099 | GFB3811_CATUST28 |
| bGMYC | 0.990099 | JEL252_CATMIN05 |
| bGMYC | 0.9899406 | PRS4514_BOMGAR01 |
| bGMYC | 0.9755248 | SCG271_SETPET04 |
| bGMYC | 0.9689109 | PRS4457_CATUST14, PRS4508_CATUST14 |
| bGMYC | 0.9584158 | GFB3797_PERCAN01, JEL249_PERCAN01, PRS4372_PERCAN01, PRS4392_PERCAN01, PRS4497_PERCAN01, SCG233_PERCAN01 |
| bGMYC | 0.9306931 | GFB3779_CNEORN01, GFB3788_CNEORN01, JEL289_CNEORN01, SCG231_CNEORN01, SCG269_CNEORN01 |
| bGMYC | 0.9198416 | GFB3848_CARPUS01, JEL232_CB1, JEL235_CB1, JEL244_CB1, JEL245_CB1, PRS4387_JUHYE04, PRS4418_CB1, PRS4476_CB1, PRS4496_CB1, PRS4517_ACAFLA04, SCG223_CB1, SCG234_CB1, SCG282_CB1 |
| bGMYC | 0.883802 | PRS4439_BT1, SCG267_PHYBOR01, SCG277_BT1, SCG280_BT1 |
| bGMYC | 0.7685941 | GFB3781_ROFI06, PRS4414_LOXLEU05, PRS4448_ROFI06, PRS4458_ROFI06 |
| bGMYC | 0.7540594 | JEL269_ACAFLA03, JEL276_ACAFLA03, PRS4456_ACAFLA03, PRS4472_ACAFLA03, PRS4482_ACAFLA03, PRS4483_ACAFLA03 |

**Table S8**. Species delimitation results from individual loci using the GMYC models. For bGMYC analyses, the number of species with posterior probability greater than 0.50 is shown.

| **Gene** | **sGMYC** | **mGMYC** | **bGMYC** |
| --- | --- | --- | --- |
| ATPase2 | 18 (6-19) | 22 (12-22) | 12 |
| RPB1 | 20 (19-21) | 21 (20-21) | 17 |
| POLD1 | 18 (7-21) | 16 (7-16) | 14 |
| KPBN1 | 21 (18-23) | 24 (19-24) | 17 |
| PRPF6 | 17 (2-20) | 16 (3-16) | 11 |
| SEC24A | 13 (6-18) | 13 (8-21) | 12 |
| SF3B1 | 12 (6-15) | 13 (5-13) | 11 |

**Table S9.** A proposed framework for future studies of avian haemosporidian species delimitation. Ideally, researchers seeking to delimit reproductively isolated units for studies of avian haemosporidian ecology and evolution will seek to collect both nuclear sequence data for coalescent species delimitation analyses and ecological host specificity data to test whether putative species have different host preferences (Box F). Alternatively, researchers may collect one of these data types, either nuclear sequence data alone (Box D and E) or host specificity data for *cytb* barcode sequences (Box C). In cases where researchers have isolated *cytb* barcodes that have no additional data (e.g. no nuclear sequence and the barcode sequences have been rarely isolated and so no estimates of host specificity can be made), haemosporidian barcodes cannot reliably be delimited as species.

|  | **Is host specificity data available?** | | |
| --- | --- | --- | --- |
|  | **No host specificity data** | **Host specificity data for one barcode** | **Host specificity data for both barcodes** |
| ***Cytb* barcode only** | [A]  No evidence available for species delimitation | [B]  No evidence available for species delimitation | [C]  Do barcodes infect significantly different host groups?   - **Evidence for species delimitation** |
| ***Cytb* and nuclear sequence data** | [D]  Are barcodes supported as independent lineages using coalescent analyses?   - **Evidence for species delimitation** | [E]  Are barcodes supported as independent lineages using coalescent analyses?   - **Evidence for species delimitation** | [F]  Are barcodes supported as independent lineages using coalescent analyses?   - **Evidence for species delimitation**   Do barcodes infect significantly different host groups?   - **Evidence for species delimitation** |
