# Supplementary material for: Integrating coalescent species delimitation with analysis of host specificity reveals extensive cryptic diversity despite minimal mitochondrial divergence in the malaria parasite genus Leucocytozoon
Source: BMC Evol Biol. 2018 Aug 30;18:128. doi: 10.1186/s12862-018-1242-x (PMC6117968; doi:10.1186/s12862-018-1242-x)
Supplement: Supplementary file 2 — Microscopic images of Leucocytozoon cytb haplotypes included in this study. This file contains microscopic images for each Leucocytozoon cytb haplotype for which material was available. (DOCX 14716 kb) [file 12862_2018_1242_MOESM2_ESM.docx]

**Additional File 2 for: “Integrating coalescent species delimitation with analysis of host specificity reveals extensive cryptic diversity despite minimal mitochondrial divergence in the malaria parasite genus *Leucocytozoon”***

**Figure S1**. Microscopic images showing morphological variation among the *cytb* haplotypes included in this study. Where available, multiple images are shown demonstrating the morphological variation within individual infections. A) *cytb* haplotypes with *Leucocytozoon* *fringillinarum* morphotypes, B) *cytb* haplotypes with *Leucocytozoon* *majoris* morphotypes, C) *cytb* haplotypes with *Leucocytozoon* *dubreuili* morphotypes.
